# Novel thin endoscope enables endoscopic submucosal dissection without retroflexion for tumor involving the whole pyloric ring

**DOI:** 10.1055/a-2344-8407

**Published:** 2024-08-07

**Authors:** Daisuke Minezaki, Teppei Akimoto, Teppei Masunaga, Naohisa Yahagi, Motohiko Kato

**Affiliations:** 1 Division of Research and Development for Minimally Invasive Treatment, Cancer Center, Keio University School of Medicine, Tokyo, Japan; 2 Center for Diagnostic and Therapeutic Endoscopy, Keio University School of Medicine, Tokyo, Japan

The narrow lumen of the pyloric ring and the duodenal bulb greatly restrict the maneuverability of an endoscope. In the forward view, the steep angle of the back side of the pyloric ring makes it difficult to approach the lesion extending to the back side of the pyloric ring 1 2 3 . Thus, retroflexion is often used during endoscopic submucosal dissection (ESD) for the lesions located there. However, it is difficult to handle the endoscope in retroflexion due to the narrow space. The recently developed therapeutic endoscope, the EG-840TP (Fujifilm, Tokyo, Japan), has the outer diameter to 7.9 mm with a downward angle of 160° ( Fig. 1 **a, b** ). Herein, we report a successful case of ESD for gastric cancer surrounding the entire pyloric ring employing this endoscope with a novel strategy ( Video 1 ).

**Fig. 1:**
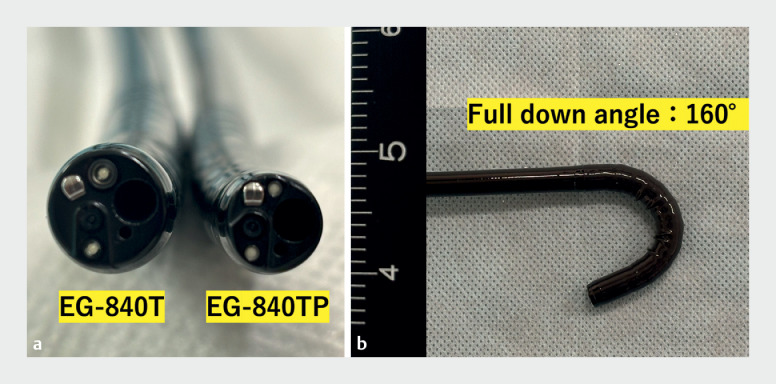
**a** Comparison of the novel thin therapeutic endoscope and normal-diameter therapeutic endoscope. Right: Novel thin therapeutic endoscope has a 7.9-mm outer diameter and 3.2-mm working channel diameter. Left: Normal-diameter therapeutic endoscope with a 9.8-mm outer diameter. **b** Novel thin therapeutic endoscope has a downward angle of 160°.

Endoscopic submucosal dissection without retroflexion using a novel thin therapeutic endoscope for gastric cancer extending from the pyloric ring to the duodenal bulb.Video 1

A 74-year-old man was referred to our hospital for endoscopic treatment. A 75-mm lesion fully encircled the pyloric ring and extended towards the duodenal bulb ( Fig. 2 **a, b** ). We used the EG-840TP and DualKnife J (Olympus Medical Systems, Tokyo, Japan). ESD was performed according to the following procedure: 1) an entire circumferential mucosal incision of the duodenal side in the forward view, 2) mucosal incision of half circumference on the lesser curvature side of the oral side, 3) creation of a submucosal tunnel penetrating from the oral side to distal side on the lesser curvature side, 4) similar creation of a tunnel on the greater curvature side. Finally, the tunnels were connected and en bloc resection was accomplished in 127 minutes without any adverse events.

**Fig. 2:**
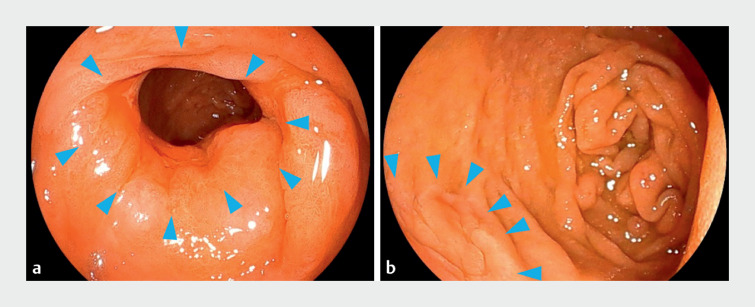
Case description. **a** The lesion was 75 mm in size and involved the entire pyloric ring. **b** The lesion extended towards the duodenal bulb.

The thinness of this endoscope reduces the maximum angle in retroflexion when the device is inserted into the endoscope. Therefore, the thinness and sharp downward angle of this endoscope provides good maneuverability and approachability in the forward view for lesions located in steep angulated and narrow spaces ( Fig. 3 ).

**Fig. 3:**
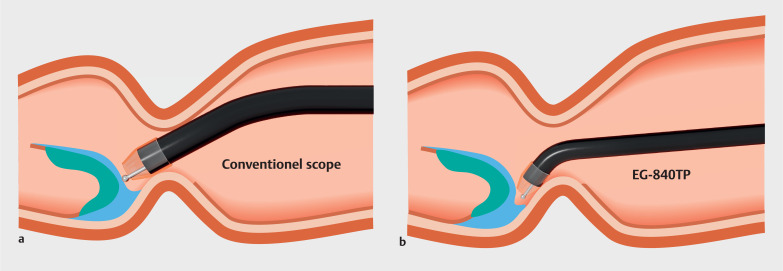
Illustration of the difference between endoscopic submucosal dissection with a conventional scope and with the novel thin therapeutic endoscope. Thanks to the sharp downward angle, retroflexion is not required during the incision and dissection from the pyloric ring to the duodenal bulb.

Endoscopy_UCTN_Code_TTT_1AO_2AG_3AD

## Notes

Endoscopy E-Videos https://eref.thieme.de/e-videos
*E-Videos* is an open access online section of the journal *Endoscopy* , reporting on interesting cases and new techniques in gastroenterological endoscopy. All papers include a high-quality video and are published with a Creative Commons CC-BY license. Endoscopy E-Videos qualify for HINARI discounts and waivers and eligibility is automatically checked during the submission process. We grant 100% waivers to articles whose corresponding authors are based in Group A countries and 50% waivers to those who are based in Group B countries as classified by Research4Life (see: https://www.research4life.org/access/eligibility/ ). This section has its own submission website at https://mc.manuscriptcentral.com/e-videos .
